# Nickel-Rich Cathodes for Solid-State Lithium Batteries: Comparative Study Between PVA and PIB Binders

**DOI:** 10.3390/molecules30142974

**Published:** 2025-07-15

**Authors:** José M. Pinheiro, Beatriz Moura Gomes, Manuela C. Baptista, M. Helena Braga

**Affiliations:** 1 Engineering Faculty, University of Porto, R. Dr. Roberto Frias s/n, 4200-465 Porto, Portugal; up202004194@up.pt (J.M.P.); up201805007@up.pt (B.M.G.); up200501276@up.pt (M.C.B.); 2 LAETA, Institute of Science and Innovation in Mechanical and Industrial Engineering, R. Dr. Roberto Frias 400, 4200-465 Porto, Portugal

**Keywords:** lithium battery, nickel rich cathode, polymer binder, solid-state electrolyte

## Abstract

The growing demand for high-energy, safe, and sustainable lithium-ion batteries has increased interest in nickel-rich cathode materials and solid-state electrolytes. This study presents a scalable wet-processing method for fabricating composite cathodes for all-solid-state batteries. The cathodes studied herein are high-nickel LiNi_0.90_Mn_0.05_Co_0.05_O_2_, NMC955, the sulfide-based electrolyte Li_6_PS_5_Cl, and alternative binders—polyvinyl alcohol (PVA) and polyisobutylene (PIB)—dispersed in toluene, a non-polar solvent compatible with the electrolyte. After fabrication, the cathodes were characterized using SEM/EDX, sheet resistance, and Hall effect measurements. Electrochemical tests were additionally performed in all-solid-state battery half-cells comprising the synthesized cathodes, lithium metal anodes, and Li_6_PS_5_Cl as the separator and electrolyte. The results show that both PIB and PVA formulations yielded conductive cathodes with stable microstructures and uniform particle distribution. Electrochemical characterization exposed that the PVA-based cathode outperformed the PIB-based counterpart, achieving the theoretical capacity of 192 mAh·g^−1^ even at 1C, whereas the PIB cathode reached a maximum capacity of 145 mAh.g^−1^ at C/40. Post-mortem analysis confirmed the structural integrity of the cathodes. These findings demonstrate the viability of NMC955 as a high-capacity cathode material compatible with solid-state systems.

## 1. Introduction

Lithium-ion batteries (LIBs) redefined energy storage, serving as the primary power source for electric vehicles (EVs) and consumer electronics [1]. Traditional LIBs are approaching their technological limit, constrained by conventional cathode active materials (CAMs) and the narrow electrochemical stability window of typical liquid electrolytes (LEs), which are also flammable [2,3]. CAMs determine the cell’s specific capacity; thus, to reach higher capacities and meet the industry’s ever-increasing demands, it is necessary to develop advanced materials with improved electrochemical and structural properties [2].

Several CAMs have been developed, with layered lithium transition metal oxides gaining popularity due to their high energy density and tunable composition, with LiNi_x_Mn_y_Co_1-x-y_O_2_ (NMC) emerging as a dominant choice in the European and American EV markets [4,5]. The synergistic effects of its three transition metals—Ni, Co, and Mn—offer the possibility to tailor its properties by adjusting their ratios: Ni enhances specific capacity, Co stabilizes the crystal structure by alleviating magnetic stresses, and Mn improves thermal and structural stability [4,6]. Driven by the demand for higher energy densities and driving ranges, the industry has progressively increased the Ni content while reducing Co, motivated not only by performance enhancement but also by ethical and geopolitical concerns surrounding Co mining [2,6,7]. This trend led to the development of high-Ni, low-Co NMC stoichiometries, from NMC622 to NMC811, and more recently NMC955 [2,6]. Nonetheless, reducing Co and Mn percentages compromises stability, limiting the upper charging voltage and cycle life [4].

Moreover, for safety reasons, LEs are being substituted by solid-state electrolytes (SSEs) [3]. Although promising for solving safety problems, namely dendrite formation and growth and solvent flammability, the use of SSEs implies the formation of solid–solid interfaces between the electrolyte itself and the electrodes of the cell, leading to high interfacial resistance and inefficient charge carrier transport [8,9,10,11]. To address these issues, several cathode fabrication strategies were developed, including the integration of SSEs within the cathode composite and the optimization of scalable fabrication methods [12,13].

Wet methods remain the most industrially viable cathode fabrication method, yet they present additional challenges when considering solid-state batteries [14]. Wet methods require the use of a binder to ensure proper electrode integrity. Conventional binders like polyvinylidene fluoride (PVDF), which is being phased out in the EU due to environmental and health concerns, are often incompatible with SSEs, particularly when processed with polar solvents that react with the electrolyte [15,16,17]. The use of compatible binder–solvent pairs that ensure the electrode’s integrity and optimized electrochemical performance of the battery is mandatory.

In this study, a scalable wet fabrication method for NMC955 cathodes using polyvinyl alcohol (PVA) and polyisobutylene (PIB) as binders and toluene as a non-polar solvent is presented. The choice of a non-polar solvent prevents spurious reactions with the chosen solid electrolyte—Li_6_PS_5_Cl (LPSCl). Synthesized cathodes underwent material characterization with scanning electron microscope (SEM), energy-dispersive X-ray spectroscopy (EDX), sheet resistance and Hall effect measurements. The resulting electrodes were then assembled into all-solid-state batteries (ASSBs) with a Li metal anode (half-cell) and underwent electrochemical characterization: potentiostatic electrochemical impedance spectroscopy (PEIS), cyclic voltammetry (CV), and galvanostatic (GCD) followed by constant voltage charge and discharge cycles. The different cell performances are compared and analyzed.

## 2. Results and Discussion

Cathodes with compositions NMC955 (57%) + LPSCl (36%) + PIB (4%) + C65 (3%) and NMC955 (57%) + LPSCl (36%) + PVA (4%) + C65 (3%) were synthesized via wet processing at 40 °C using toluene as the solvent, as detailed in Section 3. After drying, the cathodes were characterized using scanning electron microscopy/energy-dispersive X-ray spectroscopy (SEM/EDX), sheet resistance, and the Hall effect following the procedures outlined in Section 3. The cathodes were then assembled in all-solid-state cells employing LPSCl as the solid electrolyte separator and lithium metal (Li^0^) as the anode. The assembled cells underwent electrochemical characterization to evaluate their practical performance.

### 2.1. Cathode Characterization (SEM/EDX and Sheet Resistance/Hall Effect)

**Figure 1:**
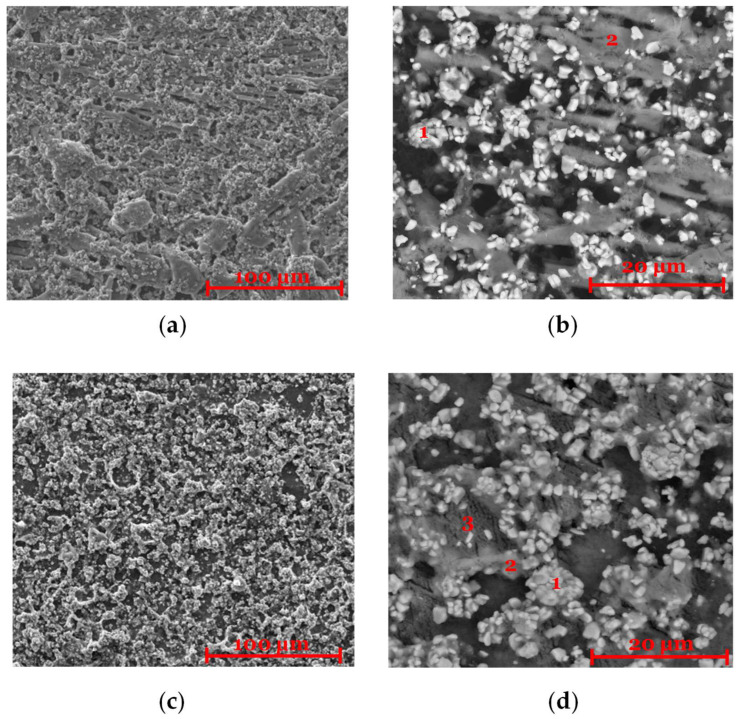
SEM/EDX acquired with a backscattered electron detector. (**a**,**b**) Cathode with composition 57% NMC955 + 36% Li_6_PS_5_Cl + 3% C65 + 4% PIB observed with 1000× and 5000× magnification. (**c**,**d**) Cathode with composition 57% NMC955 + 36% Li_6_PS_5_Cl + 3% C65 + 4% PVA observed with 1000× and 5000× magnification. Note: zones 1 and 2 in (**b**) and 1, 2, and 3 in (**d**) are analyzed in Table A2 and Table A3, respectively.

The microstructural analysis of the prepared cathode disks is presented in Figure 1. Figure 1a,b show the microstructure of the NMC955 (57%) + LPSCl (36%) + PIB (4%) + C65 (3%) composition. These images show the presence of an elastic matrix that effectively binds the cathode particles. The brighter regions, observed in Figure 1b and confirmed by elemental analysis (Table A1 and Table A2), correspond to the NMC955 active material, while the surrounding matrix consists of a mixture of LPSCl, binder, and conductive carbon. Figure 1c,d display the microstructure of the NMC955 (57%) + LPSCl (36%) + PVA (4%) + C65 (3%) composition. In this case, no elastic matrix is observed; however, the cathode remains highly homogeneous. As with the previous composition, the brighter particles correspond to the NMC955 active material, while the darker, more diffuse areas are attributed to the lighter LPSCl and binder mixture. It is highlighted that the radiation used are backscattered electrons. Elemental analysis (Table A1 and Table A2) confirms that all components are present in the expected stoichiometry. Both cathodes exhibit a uniform distribution of particles throughout the structure.

**Figure 2:**
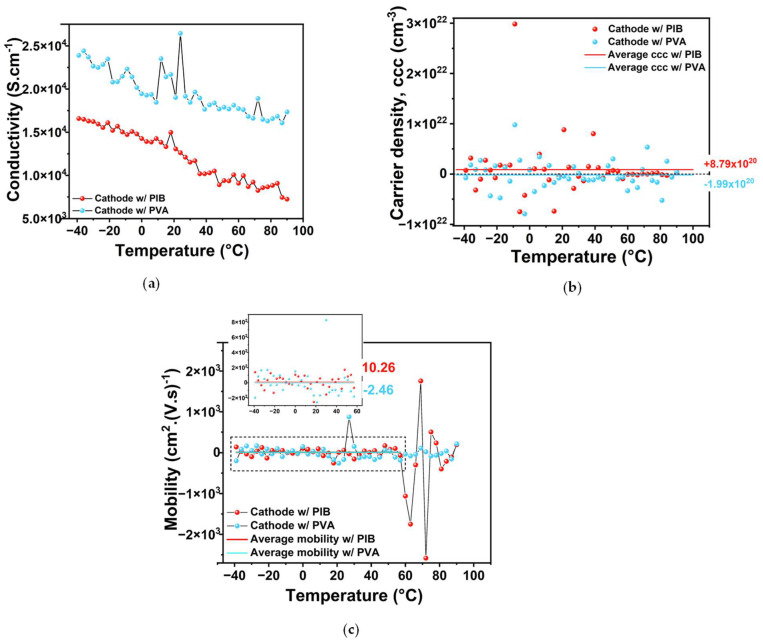
Experimental results from sheet resistance/Hall effect measurements: (**a**) Conductivity as a function of temperature—the obtained behavior is that of a typical metal; (**b**) charge carrier density as a function of temperature: positive in the cathode synthesized using PIB and negative for the cathode containing PVA; (**c**) charge carrier mobility coefficient as a function of temperature.

In both composite cathodes, NMC955 and C65 are the only intrinsically conductive components, while LPSCl exhibits semiconductor–insulator behavior and the binders (PIB or PVA) are electronic insulators. For effective battery operation, the cathode must display conductive behavior. Sheet resistance measurements reveal that both cathode compositions display an increase in resistance (a decrease in conductivity) with increasing temperature (Figure 2a), which is indicative of metallic or conductor behavior. This suggests that, despite the presence of semiconductor and insulator materials, the conductive network formed by NMC955 and C65 remains intact.

Charge carrier concentration was determined using Hall effect measurements conducted under a magnetic field of up to ±0.7 T, applied through two magnetic circuits. A Hall current of 3 mA was applied through the cathode sheets, and the Hall voltage (V_H_) and Hall coefficient (R_H_) were measured to calculate the charge carrier concentration (CCC), as detailed in Section 3. The polarity (positive or negative) of V_H_ depends on whether the charge carriers are positive or negative.

As shown in Figure 2b, the PIB-based cathode exhibited an average carrier density of 8.79 × 10^20^ cm^−3^, associated with hole-type (h⁺, positive) carriers. In contrast, the PVA-based cathode showed an average carrier density of −1.99 × 10^20^ cm^−3^, indicating electron-type (e^−^, negative) carriers (Figure 2b). The present number and polarity of average charge carriers indicate that the electrodes are degenerate semiconductors or heavily doped oxides, with partially metallic behavior. They are in the borderline between semiconductors and metallic conductors.

Mobility analysis (Figure 2c) reveals a transition region for the PIB-based cathode between 60 and 80 °C, characterized by a sudden increase in negative charge mobility followed by alternating peaks of positive and negative carrier mobility. The average charge mobility between −40 and 60 °C is 10.26 cm^2^·V^−1^·s^−1^ for the PIB-based cathode and −2.46 cm^2^·V^−1^·s^−1^ for the PVA-based cathode, showing once again that PIB and PVA binder-based cathodes are more prone to display positive and negative charge mobility, respectively, likely indicating that the PIB and PVA are, respectively, better cationic and electronic conductors. The change in charge mobility observed in the PIB-based cathode around 60–80 °C (Figure 2c) is likely due to thermal softening, which can change its mechanical properties. As PIB transitions into a viscoelastic state, conductive pathways may be compromised by weakening contact between the active materials. Above −70 °C, PIB is a supercooled liquid. Above ≈200 °C, depending on the type of PIB (for example, from its molecular weight), it melts.

The results obtained for conductivity and CCC for PIB are consistent with those reported in [18] for SBR binder-based NMC955 cathode (1.13 × 10^21^ h^+^ cm^−3^). The conductivity shows the same trend, decreasing with temperature, and the measured values are of the same order of magnitude. These findings reinforce the potential of NMC955 as a promising high-capacity material, particularly due to its compatibility with various alternative binders that are more sustainable than conventional options such as PVDF.

### 2.2. Cathode Electrochemical Characterization (PEIS, CV, and GCD)

All-solid-state coin cells were assembled with the obtained cathode disks, LPSCl as the electrolyte–separator and Li metal as the anode. A summary of all fabricated cells can be found in Table A1.

**Figure 3:**
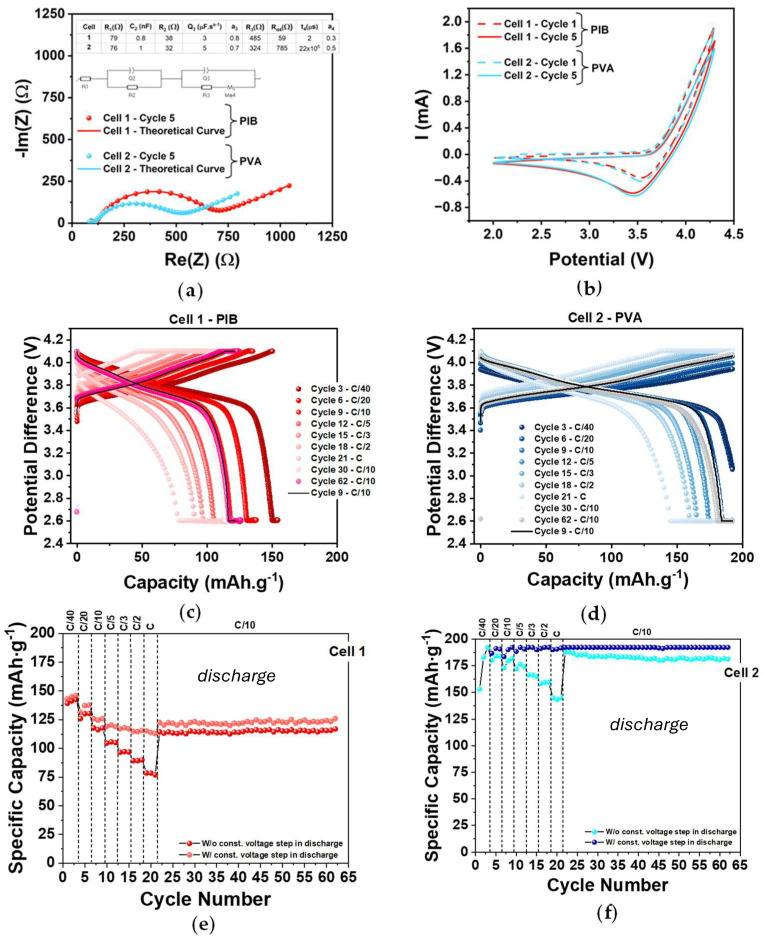
Experimental results from electrochemical characterization: (**a**) PEIS analysis—both cells were modeled using the same equivalent circuit. The presented cycle is the last one before CV; (**b**) CV diagrams from each cell, cycles 1 and 5, are presented, displaying a stable behavior from start to finish of the test; (**c**) GCD test performed on Cell 1 (PIB binder), the third and last cycle for each C-rate is plotted; (**d**) GCD test performed on Cell 2 (PVA binder), the third and last cycle for each C-rate is plotted; (**e**) specific discharge capacity as a function of number of cycles from Cell 1 (PIB binder); (**f**) specific discharge capacity as a function of number of cycles from Cell 2 (PVA binder).

After assembly, cells underwent PEIS and CV tests. The equivalent circuit derived from the PEIS analysis was used to determine the ionic behavior of the system, with component values extracted accordingly (Figure 3a). R1, an ohmic resistance, corresponds to the conduction of the most mobile ionic species at high frequencies and is associated with interfacial contact resistance; ideally, it should approach zero. R2, representing the diameter of the first semicircle in the Nyquist plot, models bulk ionic transport through the electrolyte via ion hopping. This process is effectively represented by a resistor (R2) in parallel with a capacitor, capturing the dielectric behavior of the solid electrolyte in an “ideal” capacitor of thickness *d*. It does not account for the movement of the ions at the interfaces. In solid-state inorganic electrolytes, Li⁺ migration in one direction is accompanied by the movement of negatively charged vacancies in the opposite direction, influencing both electrodes simultaneously, and therefore the corresponding resistance is associated in parallel with the “ideal” capacitor. Interfacial processes, particularly those related to electrical double layer formation, are reflected in the elements Q3, R3, and Rd4. These elements represent non-ideal Li⁺ diffusion within the double-layer structure, mainly occurring at the positive electrode and limited to a narrow region with unknown thickness, usually of the order of angstrom.

This interpretation applies to both Cell 1 and Cell 2 (Figure 3a). In both cases, R1 and R2 exhibit low and comparable values, indicating similar bulk conductivity. A second semicircle is clearly visible in both spectra, signifying the formation of a double layer at the electrode–electrolyte interface. Notably, R3 is higher in the PIB cell, suggesting increased resistance to interfacial charge transfer or Li⁺ accumulation. Additionally, both PEIS spectra exhibit a linear low-frequency tail, characteristic of diffusion-controlled processes, confirming the presence of diffusion behavior.

The CVs show a reduction peak at approximately 3.5 V while discharging the cell (Figure 3b). The current response rises above 4.1 V, suggesting a shift in charge carrier origin from e^−^ to h^+^ (with possible loss of O^2−^), showcasing the importance of not charging beyond this cut-off voltage. The used voltage window was verified in already published theoretical simulations [18]. All CV curves are directly comparable since the active area is the same for all cells (≈0.5 cm^2^) and the mass of active material is similar (Table A1). For both cathode formulations the maximum current is situated at approximately 1.8 mA, showing very similar behavior.

All cells were subjected to electrochemical cycling between 2.6 and 4.1 V at various C-rates (C/40, C/20, C/10, C/5, C/3, C/2, and C), Figure 3c,d. Tests were conducted at room temperature without the application of external pressure. To prevent structural degradation of the NMC955 material due to complete lithium extraction, only approximately 70% of the lithium ions were targeted for de-lithiation. This limitation reduces the theoretical specific capacity of the NMC955 cathode to an estimated 192 mAh·g^−1^.

**Figure A1:**
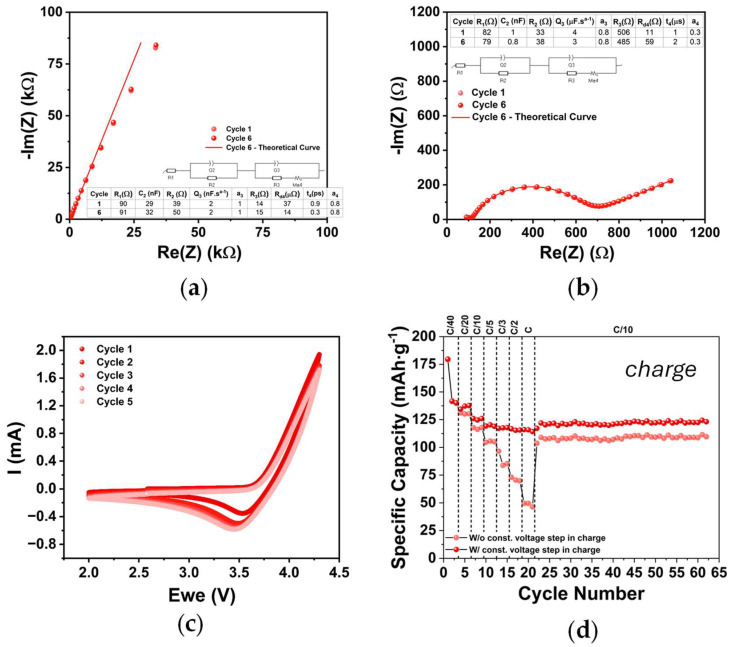
Electrochemical characterization of Cell 1 (PIB-based): (**a**) PEIS analysis before CV; (**b**) PEIS analysis after CV; (**c**) CV results, all cycles; (**d**) specific charge capacity as a function of number of cycles.

**Figure A2:**
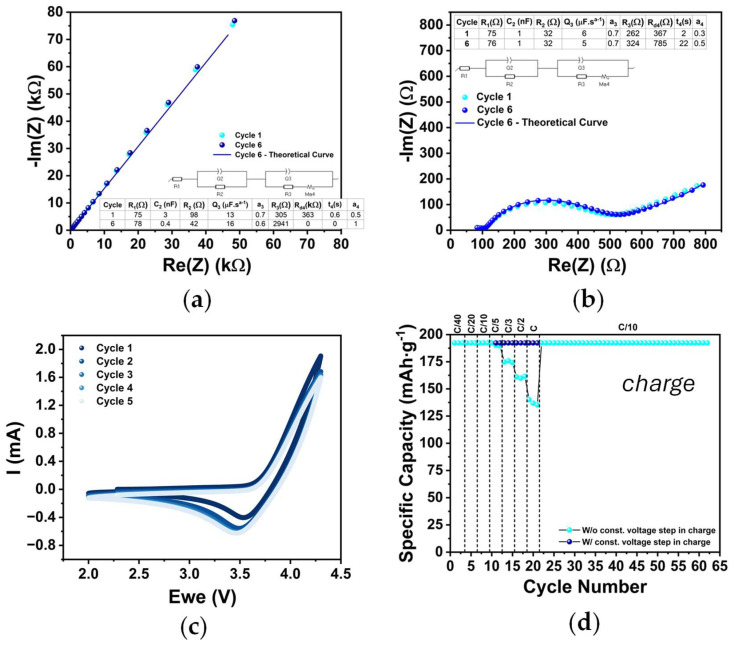
Electrochemical characterization of Cell 2 (PVA-based): (**a**) PEIS analysis before CV; (**b**) PEIS analysis after CV; (**c**) CV results, all cycles; (**d**) specific charge capacity as a function of number of cycles.

**Figure A3:**
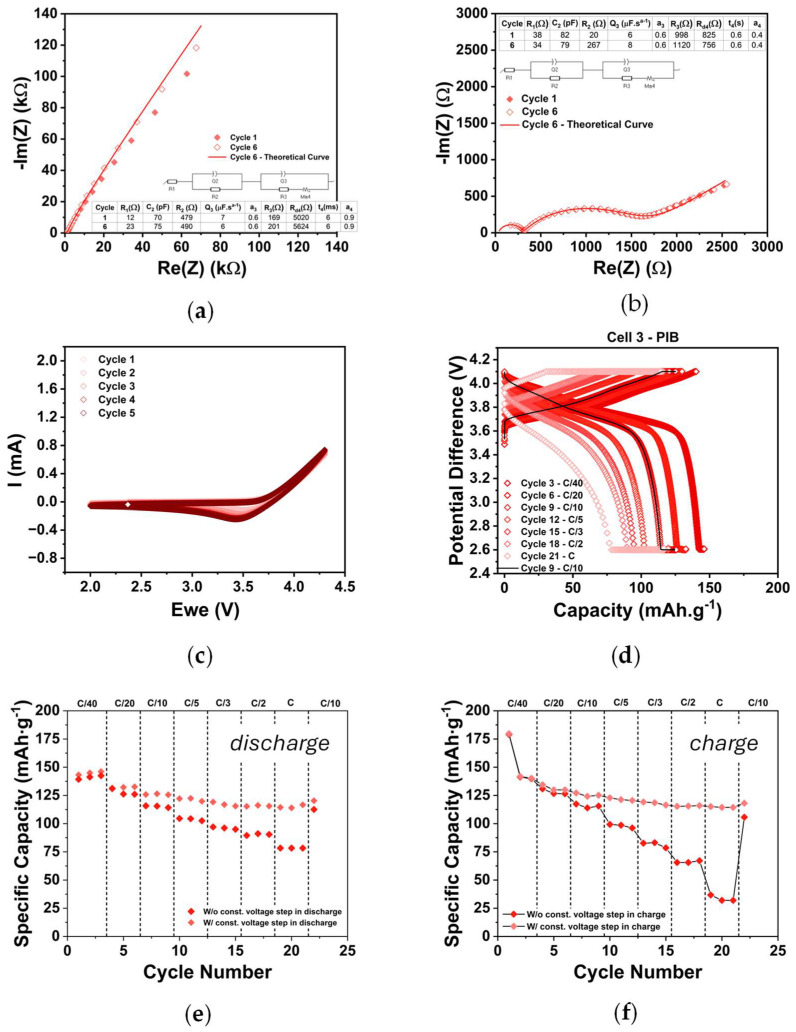
Electrochemical characterization of Cell 3: (**a**) PEIS analysis before CV; (**b**) PEIS analysis after CV; (**c**) CV results, all cycles; (**d**) GCD results, the third and last cycle at each C-rate is plotted; (**e**) specific discharge capacity as a function of cycle number; (**f**) specific charge capacity as a function of cycle number.

**Figure A4:**
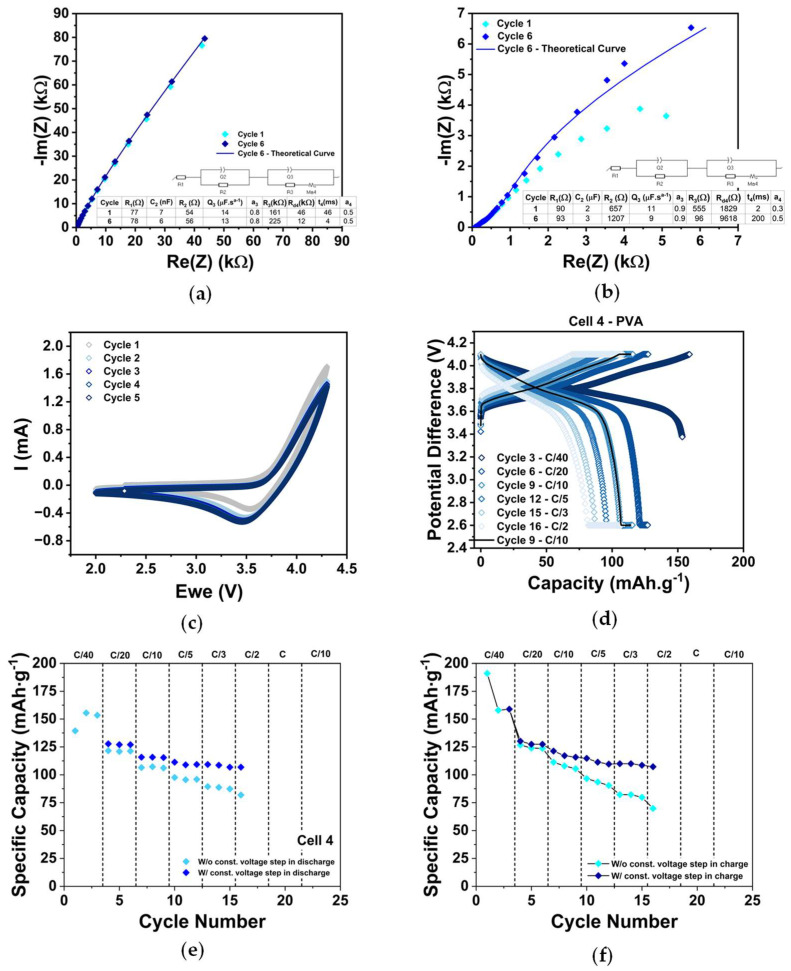
Electrochemical characterization of Cell 4: (**a**) PEIS analysis before CV; (**b**) PEIS analysis after CV; (**c**) CV results, all cycles; (**d**) GCD results, the third and last cycle at each C-rate is plotted; (**e**) specific discharge capacity as a function of cycle number; (**f**) specific charge capacity as a function of cycle number.

The electrochemical performance of the best-performing cell for each binder formulation is presented in Figure 3c,d. For the PIB-based cell, a maximum capacity of approximately 145 mAh·g^−1^ was achieved at C/40, with capacity decreasing as the C-rate increased (Figure 3e). The theoretical capacity was not reached at any point during cycling. In contrast, the PVA-based cell reached the theoretical capacity multiple times, even without requiring a constant voltage step, for lower C-rates. For C/40, the theoretical capacity was achieved before reaching the upper cut-off voltage of 4.1 V. Although the capacity decreased with increasing C-rate, the cell maintained a minimum capacity of 150 mAh·g^−1^ at 1C, and with the addition of a CV step, it was able to reach the theoretical capacity (Figure 3f). Furthermore, upon repeating the C/10 rate, the PVA-based binder cell demonstrated a higher capacity than at the beginning of cycling, suggesting ongoing improvement in electrochemical behavior (Figure 3f). Additional experimental data supporting these conclusions is presented in the Figure A1 and Figure A2. Figure A3 and Figure A4 present experimental results for Cell 3 and Cell 4 (Table A1) with the same cathode formulations, showing comparable performance to the results reported in the main text. The electrochemical performance achieved shows that both cathodes can be used to fabricate stable, long-lasting cells with promising discharge capacities.

### 2.3. Post-Mortem Characterization (SEM/EDX)

**Figure 4:**
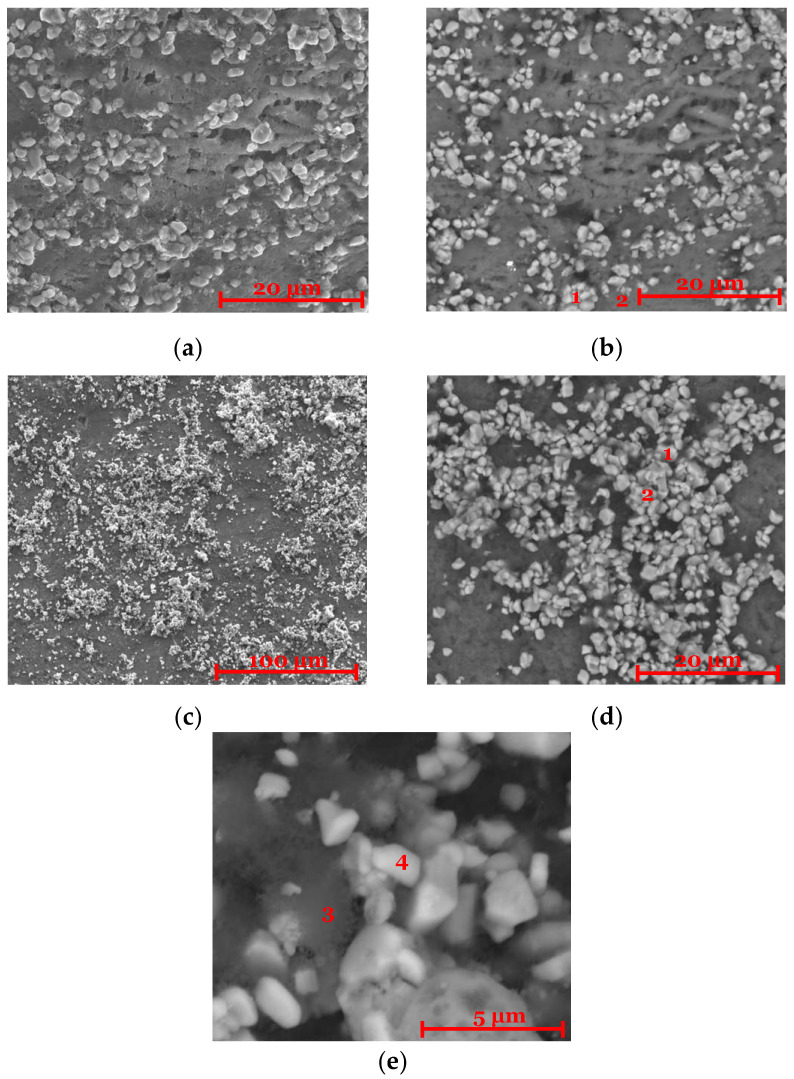
Cathode characterization. Images were acquired with SEM/EDX and a backscattered electron detector. (**a**,**b**) Cathode with composition 57% NMC955 + 36% Li_6_PS_5_Cl + 3% C65 + 4% PIB observed with 5000× magnification; (**c**–**e**) cathode with composition 57% NMC955 + 36% Li_6_PS_5_Cl + 3% C65 + 4% PVA observed with 1000×, 5000×, and 20,000× magnification. Note: zones 1 and 2 in (**b**), 1 and 2 in (**d**), and 3 and 4 in (**e**) are analyzed in Table A4 and Table A5.

Once the GCD tests were finished, cells were opened inside the glove box, and the cathodes were analyzed using post-mortem SEM/EDX. The performance of the cells selected for this analysis is shown in Figure A3 and Figure A4. In the PIB-based cathode, remnants of the elastic matrix were still visible (Figure 4a,b). A decrease in sulfur content compared to the initial composition (Table A2 and Table A4) was observed, consistent with the formation of sulfates as reported in [18]. NMC955 particles remained detectable, but a reduction in oxygen content was noted (Table A2 and Table A4), likely due to oxygen release during charging. This observation is supported by the CV data, which showed increased current near 4.1 V, suggesting the occurrence of competing side reactions.

For the PVA-based cathode (Figure 4c–e), a similar reduction in oxygen content was observed (Table A3 and Table A5). While Figure 4c seems to show fewer deposited particles compared to Figure 1c, this comparison should be interpreted with caution. The more difficult demolding process for the PVA sample may have damaged the surface, potentially affecting the observed microstructure and the quality of the analysis.

## 3. Materials and Methods

Solubility tests were conducted to assess the viability of pairing PVA [HiMedia, Poly (vinyl alcohol) cold water soluble] and PIB (Sigma Aldrich, St. Louis, MO, USA, BM, USA Polyisobutylene) with toluene (Honeywell, purity ≥ 99.7%). A fixed mass of PVA was added to 5 mL of toluene and stirred magnetically at 250 rpm for 24 h. Experiments were conducted at both room temperature and 40 °C. The results indicated that PVA was miscible with toluene at the elevated temperature of 40 °C, even having higher polarity. To enable a direct comparison between PVA and PIB as binder materials, cathode synthesis was performed at 40 °C for both materials.

The composite cathode material was prepared using a mixture of 57% NMC 955 (Huayou New Energy Technology, Quzhou, China), 36% Li_6_PS_5_Cl (NEI Corporation, Somerset, NJ, USA), 3% C65 (TIMCAL graphite and carbon super C65), and 4% binder: PVA or PIB. The used solvent was toluene (Honeywell International Inc., Charlotte, NC, USA, purity ≥ 99.7%), and the target for the total mass of the cathode was 1 g.

The binder was initially mixed with 2 mL of toluene and magnetically stirred. The active material and conductive agent were then added together, followed lastly by the solid electrolyte powder. Each mixing step was performed for 24 h at 250 rpm at 40 °C. To ensure a proper mixing and a homogeneous deposition, an additional 0.5 mL of toluene was added with the electrolyte at the last stirring stage. Once a homogeneous slurry was obtained, it was deposited with a thickness of 250 µm onto pre-heated aluminum foil coated with carbon (MSE Supplies LLC, Tucson, AZ, USA) of 15 µm thickness and left to dry overnight. The dried cathode film was then cut into 10 mm diameter disks using a TMAX-CN cutter. All procedures were conducted inside a GS Glovebox (O_2_ < 1 ppm, H_2_O < 1 ppm).

A scanning electron microscope (SEM) (FEI QUANTA 400 FEG ESEM, Hillsboro, OH, USA) was used for microstructural analysis. Chemical composition was determined using an EDAX Genesis X4M energy-dispersive X-ray spectrometer (EDX) (Thermo Scientific, Waltham, MA, USA). Observations were carried out at an accelerating voltage of 15 kV, with a spot size between 3 and 5 and a working distance of 10 µm. The microstructure was observed with magnifications ranging from 20× to 20,000× using a backscattered electron detector (Thermo Scientific, Waltham, MA, USA).

Hall effect measurements were conducted using the Linseis HCS 1 system, equipped with two neodymium magnetic circuits on a movable sled. Samples were mounted with edge contacts, and a perpendicular magnetic field of ±0.7 and 0 T was applied. A constant current of 3 mA was applied to the samples (NMC955 cathodes with PIB or PVA), and the resulting Hall voltage ($V_{H}$), generated orthogonal to both the current (I) and magnetic field (B), was precisely measured with high sensitivity from −40 to 90 °C. Each temperature is equilibrated for a heating/cooling rate of ≈0.5 °C.min^−1^. The Hall coefficient ($R_{H}$) and charge carrier concentration (n) can be calculated using the equations $R_{H}=\frac{V_{H}\cdot d}{IB}$, where d is the sample thickness, and $n=\frac{1}{e\cdot R_{H}}$.

Sheet resistance analysis was performed on the electrodes using the Van der Pauw method with four collinear contacts. Current and voltage were applied across different contact pairs in multiple configurations to ensure accuracy. Sheet resistance $R_{S}$ was determined using the formula $R_{S}=\frac{V}{I\cdot f}$, with correction factor f, and the Van der Pauw relation $e^{-\pi R_{s1}/R_{s}}+e^{-\pi R_{s2}/R_{s}}=1$. All tests were performed under controlled conditions, with the chamber evacuated to 10^−2^ bar and flushed with nitrogen gas (4–5 L/min) to reduce noise, temperature fluctuations, and unwanted reactions.

To assemble the cells, 100 mg of Li_6_PS_5_Cl were pressed onto the cathode disk for 90 s at 1 ton, using a mold with a 10 mm diameter and the SPECAC press machine, and forming a bilayer. The coin cells were fabricated by stacking the anode on top of the SSE sheet of the bilayer, followed by a spacer and a spring. The used anode material was Li metal with a thickness of 200 μm and an 8 mm diameter. The cells were closed using a pressure-controlled electric crimper for coin cells MSK-160E. The capacities of the cathodes were calculated based on the active area of the cathode, which is constrained by the size of the anode (d ≈ 8 mm), and considering the theoretical capacity of 192 mAh.g^−1^. The anode was made smaller than the cathode to avoid short circuits by possible contact at the disk’s edge. Three types of electrochemical tests were performed to evaluate the fabricated cells. Potentiostatic electrochemical impedance spectroscopy (PEIS) tests were performed to evaluate the internal impedance. Cyclic voltammetry (CV) tests were performed to determine the capacity of the obtained cell. Both were performed using a Biologic VMP-300 potentiostat (Seyssinet-Pariset, France). Lastly, galvanostatic charge–discharge (GCD) was implemented to evaluate the cyclability and behavior of cells at different C-rates. The latter were performed in a Neware-CT-4008Tn-5V-20mA battery tester (Montclair, CA, USA) with 2.6 and 4.1 V cut-off voltages.

## 4. Conclusions

This work demonstrates a scalable and cost-effective fabrication method for an Li^0^/Li_6_PS_5_Cl/NMC955 all-solid-state half-cell. The high specific capacities achieved are attributed to the high-Ni, low-Co stoichiometry of NMC955, which enhances energy density. Both binders, PVA and PIB, emerge as viable alternatives to the conventional PVDF. PVA shows itself as the best-performing binder, which is likely associated with an average excess of negative charges ($\bar{e}$) of 2.4 mAh.g^−1^, corresponding with higher electronic and ionic conductivity, while the PIB shows a correspondent excess of positive charges ($h^{+}$ or ${Li}^{+}$ in the cathode) of 10.7 mAh.g^−1^, considering the densities of the cathodes of 3.64 g.cm^−3^ (PIB-based) and 3.73 g.cm^−3^ (PVA-based). PVA is the better choice for ionic conduction. Neither PVA nor PIB is naturally good at electronic conduction, but PVA is more efficient when composited with conductive fillers (e.g., carbon, graphene) to form mixed-conduction systems.

Cathodes synthesized with the proposed method deliver stable and long-lasting performance, achieving capacities of 145 mAh·g^−1^, at C/40 in the case of the PIB formulation and 192 mAh.g^−1^ at all the tested C-rates for the PVA formulation. Cells maintained a high-rate capability at C/10 after being tested from C/40 to 1C. Electrochemical testing, alongside SEM/EDX characterization and Hall effect/sheet resistance measurements, highlights the role of cathode design in determining overall cell performance and cycle life.

## Data Availability

The data will be made available upon reasonable request.

## Abbreviations

The following abbreviations are used in this manuscript:

ASSB	all-solid-state battery
CAM	cathode active material
CV	cyclic voltammetry
EDX	energy-dispersive X-ray spectroscopy
EV	electric vehicle
GCD	galvanostatic charge and discharge
LE	liquid electrolyte
LIB	lithium-ion battery
LPSCl	Li_6_PS_5_Cl
NMC	LiNi_x_Mn_y_Co_1-x-y_O_2_
PEIS	potentiostatic electrochemical impedance spectroscopy
PIB	polyisobutene
PVA	polyvinyl acetate
PVDF	polyvinylidene fluoride
SEM	scanning electron microscope
SSE	solid-state electrolyte

## Appendix A

**Table A1:** Summary table of the cells reported in the paper.

Cell ID	Binder	Mass of NMC 955 (mg)	Areal Capacity (mAhcm^−2^)	Correspondent Figures
Cell 1	PIB	438	0.168	Figure 1a,b, Figure 2, Figure 3a–c,e and Figure 4a,b
Cell 2	PVA	182	0.07	Figure 1c,d, Figure 2, Figure 3a,b,d,f and Figure 4c–e
Cell 3	PIB	438	0.168	Figure A3
Cell 4	PVA	292	0.112	Figure A4

**Table A2:** EDX data for the PIB-based cathode (57% NMC955 + 36% Li_6_PS_5_Cl + 3% C65 + 4% PIB) shown in Figure 1.

At %								
	C	O	Ni	Mn	Co	P	S	Cl
1	4.29	54.24	34.54	1.70	1.49	0.29	2.61	0.84
2	12.15	28.47	2.15	0.14	0.28	8.45	41.99	6.37

**Table A3:** EDX data for the PVA-based cathode (57% NMC955 + 36% Li_6_PS_5_Cl + 3% C65 + 4% PVA) shown in Figure 1.

At %								
	C	O	Ni	Mn	Co	P	S	Cl
1	5.00	44.67	38.52	1.93	2.01	0.90	6.22	0.75
2	8.78	3.00	66.91	1.33	2.20	2.61	13.23	1.94
3	13.19	19.15	2.65	0.44	0.50	7.66	47.40	9.01

**Table A4:** *Post-mortem* EDX data for the PIB-based cathode (57% NMC955 + 36% Li_6_PS_5_Cl + 3% C65 + 4% PIB) shown in Figure 4.

At %								
	C	O	Ni	Mn	Co	P	S	Cl
1	9.01	47.69	37.68	1.69	1.87	0.33	1.40	0.33
2	24.28	25.87	1.12	0.22	0.26	8.10	33.82	6.33

**Table A5:** *Post-mortem* EDX data for the PVA-based cathode (57% NMC955 + 36% Li_6_PS_5_Cl + 3% C65 + 4% PVA) shown in Figure 4.

At %								
	C	O	Ni	Mn	Co	P	S	Cl
1	51.29	25.60	17.37	0.88	1.48	0.23	2.90	0.25
2	7.22	52.53	34.18	1.55	1.81	0.12	2.43	0.16
3	71.14	11.98	11.56	0.67	0.79	0.43	3.00	0.43
4	13.68	50.34	30.15	1.47	1.51	0.38	2.17	0.30
